# Nisin and ε-polylysine combined treatment enhances quality of fresh-cut jackfruit at refrigerated storage

**DOI:** 10.3389/fnut.2024.1299810

**Published:** 2024-02-14

**Authors:** Liping Zeng, Aiping Fan, Guangming Yang, Yuping Nong, Yifan Lu, Ruopeng Yang

**Affiliations:** ^1^College of Chemistry and Resources Engineering, Honghe University, Mengzi, China; ^2^Yunnan Province International Joint Laboratory of Green Food, College of Chemistry and Resources Engineering, Honghe University, Mengzi, Yunnan, China

**Keywords:** fresh-cut jackfruit, nisin, ε-polylysine, storage quality, microorganisms

## Abstract

This study investigated the effects of nisin combined with ε-polylysine on microorganisms and the refrigerated quality of fresh-cut jackfruit. After being treated with distilled water (control), nisin (0.5 g/L), ε-polylysine (0.5 g/L), and the combination of nisin (0.1 g/L) and ε-polylysine (0.4 g/L), microporous modified atmosphere packaging (MMAP) was carried out and stored at 10 ± 1°C for 8 days. The microorganisms and physicochemical indexes were measured every 2 days during storage. The results indicated that combined treatment (0.1 g/L nisin, 0.4 g/L ε-polylysine) had the best preservation on fresh-cut jackfruit. Compared with the control, combined treatment inhibited microbial growth (total bacterial count, mold and yeast), reduced the weight loss rate, respiratory intensity, polyphenol oxidase and peroxidase activities, and maintained higher sugar acid content, firmness, and color. Furthermore, it preserved higher levels of antioxidant compounds, reduced the accumulation of malondialdehyde and hydrogen peroxide, thereby reducing oxidative damage and maintaining high nutritional and sensory qualities. As a safe application of natural preservatives, nisin combined with ε-polylysine treatment has great application potential in the fresh-cut jackfruit industry.

## 1 Introduction

Jackfruit (*Artocarpus heterophyllus* Lam.), commonly called “vegetarian’s meat,” is currently recognized as the largest tree-born tropical and subtropical fruit on earth (1, 2). Its golden color, sweetness, aromatic scent, and juiciness appeal to consumers incredibly. Furthermore, jackfruit holds considerable nutritional and medicinal value, containing ample amounts of carbohydrates, proteins, fruit fiber, minerals, vitamins, and bioactive substances (3). Notably, jackfruit is characterized by its large size and thick peel, with a relatively low edible part of 25–35%, making it difficult for ordinary consumers to assess the maturity and open the fruit (4). In addition, its high sugar content poses challenges for storage, and the sale of the whole fruit is prone to expensive transportation costs and environmental problems, including waste of resources, thus violating the principles of sustainable development (5). As a minimal processing method, fresh-cut processing not only meets the demand for nutrition, health, and convenience but also improves the utilization rate of jackfruit waste resources and extends the industrial chain (6, 7). However, after washing, peeling, and cutting processes, there will be an increase in the risk of spoilage microbial infection and rapid quality deterioration of fresh-cut products (8). The loss of natural protective barrier due to mechanical cutting and other operations during the processing of jackfruit results in tissue damage, nutrient leakage, accelerated physiological and biochemical changes, including the increase of microorganisms such as bacteria (*Pantoea agglomerans* and *Pseudomonas fluorescens*), mold and yeast (9, 10). These conditions will seriously affect the quality and shelf life of fresh-cut jackfruit. Therefore, seeking safe, efficient, and green preservation techniques is necessary to improve fresh-cut jackfruit quality and microbiological safety.

Chemical preservatives are a well-established technology for controlling food spoilage caused by microbial contamination (11). With the deepening of the green consumption concept, consumers have increasingly requested fresher, safer, and longer shelf-life foods (12). Therefore, natural preservatives instead of chemical preservatives have great market potential. Nisin is a water-soluble, heat-stable natural antibacterial peptide produced by *Lactococcus lactis* (13). Since 1988, nisin has been awarded a “generally recognized as safe (GRAS)” substance by the United States Food and Drug Administration (FDA) (14) and has been approved for food preservation by more than 50 countries, including the United States and China. Nisin has strong antibacterial properties against Gram-positive bacteria. However, it has a poor inhibitory effect on Gram-negative bacteria, mold and yeast, and is often used in combination with other antibacterial agents (15, 16). ε-polylysine (ε-PL) is a cationic polypeptide produced by *Streptomyces albulus* that is water-soluble, thermally stable, and non-toxic (17, 18). ε-PL is a broad-spectrum bacteriostatic agent that meets food safety requirements without affecting taste and improves fruit disease resistance (19, 20). ε-PL has been approved by the FDA as a safe food additive since 2003 and has been used in various food preservation applications in various countries (21). Both nisin and ε-PL are natural food preservatives, and previous studies (22) have confirmed that a single nisin, a single ε-PL, and a combined treatment significantly affect food preservation. However, their effectiveness is influenced by various factors, such as the type of food, duration of inhibitor action, and concentration and ratio of nisin and ε-PL. Multiple studies have reported the application of nisin or ε-PL in preserving whole (15) and fresh-cut products, including iceberg lettuce (23), potato (24), and kiwifruit (25). Nevertheless, the application of nisin and ε-PL combined treatment in fresh-cut fruits and vegetables is relatively rare. The aim of this study was to evaluate the effect of nisin and ε-PL combined treatment on microorganisms and quality of fresh-cut jackfruit to develop a suitable fresh-cut jackfruit composite natural preservative as an alternative to chemical preservatives.

## 2 Materials and methods

### 2.1 Materials and reagents

Plate count agar (PCA) and *Bengal red medium* were purchased from Beijing Luqiao Technology Co., Ltd. (Beijing, China). Nisin (food grade) and ε-PL (food grade) were purchased from Zhengzhou Bainafo biological Co., Ltd. (Zhengzhou, China). Gallic acid, polyvinylpyrrolidone, and guaiacol were purchased from Aladdin Reagent Co., Ltd. (Shanghai, China). 3, 5-dinitrosalicylic acid (DNS) was purchased from Xiamen Haibiao Technology Co., Ltd. (Xiamen, China). Other agents of analytic grade were purchased from Sinopharm Chemical Reagent Co., Ltd. (Beijing, China).

### 2.2 Raw materials

Fresh jackfruit was picked from the Pingbian jackfruit planting base in Yunnan, China, and transported to the laboratory on the day of picking. The eight-ripe whole jackfruit fruits (Total soluble solids content: 22–24%) were pre-cooled at 10 ± 1°C for 24 h. The whole jackfruit and knives were cleaned and disinfected with 0.02% (w/v) sodium hypochlorite solution. Under clean conditions, cut along the main axis of jackfruit and take out the core to select jackfruit pulps with uniform size, no pests, diseases, and mechanical damage.

### 2.3 Preparation of natural preservative solutions and screening for the ratio of nisin and ε-PL combined treatment

According to previous literature (26, 27). nisin or ε-PL was applied more in the concentration range of 0–0.6 g/L in fresh-cut fruits and vegetables, and the preservation effect was directly proportional to the concentration within a certain range, but the effect is not significant if the concentration is too high. It was determined that 0.5 g/L natural preservative solutions were used for fresh-cut jackfruit preservation through previous literature and preliminary experiments. In this experiment, we compared the use of (0.5 g/L) and ε-PL (0.5 g/L) as individual biological preservatives, as well as a combined treatment (0.5 g/L) prepared at different ratios of nisin to ε-PL, specifically 1:1, 1:2, 1:3, 1:4, and 1:5 (v/v). We measured total bacterial count at 0, 4, and 8 days after the samples were stored at 10 ± 1°C. The combined treatment that showed the most effective antimicrobial properties was selected for subsequent preservation experiments.

### 2.4 Sample treatments

The screened raw materials were randomly divided into four groups and treated as follows: (1) distilled water (CK); (2) 0.5 g/L nisin solution (Nisin); (3) 0.5 g/L ε-PL solution (ε-PL); (4) nisin (0.1 g/L) and ε-PL (0.4 g/L) combination solution (Nisin+ε-PL). The samples were immersed in the above solutions for 5 min at a ratio of 1:20 (w/w), then drained by cold air, and placed in polypropylene boxes (PP) sealed with polypropylene preservative film (PP, thickness: 70 μm, pore size: 30 μm) microporous modified atmosphere packaging (MMAP). Each box contains approximately 7–8 fruit pulps with a mass control of 200 ± 10 g. Then store the sample at 10 ± 1°C for 8 days, and measure the indicators every 2 days. Normally, the suitable controlled atmosphere range for most fresh-cut produce is an oxygen concentration of 1–6% and a carbon dioxide concentration of 1–6%. After the pre-experimental screening, the optimal MMAP conditions for fresh-cut jackfruit are 5% O_2_, 6% CO_2_, and 15 holes are used for packaging.

### 2.5 Microorganisms

The total bacterial count, mold and yeast were determined according to Chinese standards GB4789.2 and GB4789.15. Approximately 25 g sample was homogenized with 225 mL sterile 0.9% (w/v) NaCl solution, then the resulting suspension was gradient-diluted in sterile 0.9% NaCl solution (1:10, v/v), 1 mL diluent was mixed with PCA *medium* and cultured at 37°C for 48 h to determine the total bacterial count. The 1 mL diluent was mixed with *Bengal red medium* at 28°C for 96 h to determine mold and yeast. Both results were expressed as lg CFU/g.

### 2.6 Physicochemical parameters

#### 2.6.1 Color

The color was analyzed using a colorimeter (WSC-S, Shanghai Precision Instrument Co., Ltd., Shanghai, China) on the surface of three random jackfruit pulp. Each sample was measured five times in parallel and obtained parameters: L* (light/dark), a* (red/green), b* (yellow/blue), and color difference (∆E). Color difference (ΔE) was performed according to the following equation:

$$\Delta E=\sqrt{\Delta L^{2}+\Delta a^{2}+\Delta b^{2}}$$

#### 2.6.2 Weight loss

Weight loss was estimated using a gravimetric method as follow:

$$W\mathrm{eight}\;\mathrm{loss}\;\left(\%\right)=\left(m_{1}-m_{2}\right)/m_{1}\times 100$$

where m_1_ is the initial weight and m_2_ is the sampling point weight.

#### 2.6.3 Firmness

Firmness was measured on five random samples by a texture analyzer (TA.new plus, ISENSO, United States) equipped with a diameter of 2 mm. The samples were measured near the equator using specific parameters: a test speed of 2 mm/s, a penetration depth of 3 mm, a trigger force of 10 g, and a spacing of greater than 5 mm.

#### 2.6.4 Total soluble solids

Total soluble solids (TSS) content was obtained with a portable refractometer (LB90A Guangzhou Mingrui Electronic Technology Co., Ltd., China). Approximately 20 g sample from each treatment group was randomly weighed, homogenized, and filtered through three layers of clean gauze.

#### 2.6.5 Total acidity

Total acidity (TA) content was determined by homogenizing and mixing a 5 g sample with distilled water (1:20, w/v) for 30 min and then filtering. The 20 mL filtrate containing 1% phenolphthalein indicator received titration with 0.01 mol/L NaOH.

#### 2.6.6 Reducing sugar

The determination method of reducing sugar was derived from the previous experimental method (28). In short, 0.5 g sample was homogenized in 10 mL distilled water and incubated at 80°C for 30 min. After cooling, the mixture was centrifuged (10,000 r/min, 20 min, 4°C) to obtain the supernatant. 0.5 mL supernatant was added with distilled water to 2 mL, mixed with 1.5 mL 3,5-dinitro salicylic acid reagent thoroughly, heated in a boiling water bath for 5 min, and measured the absorbance was at 540 nm. Reducing sugar content was calculated by the glucose standard curve. The standard curve was shown in Supplementary Figure S1, and the equation of the standard curve was y = 0.0047x−0.3148.

#### 2.6.7 Respiration rate

The respiration rate was recorded with a fruit and vegetable respirometer (SY-1022, Shijiazhuang Shiya Technology Co., Ltd., Hebei, China). Two jackfruits were randomly selected from different treatment groups, respectively, and placed in a Φ 60*90 mm breathing chamber for 1 h. The result of respiratory intensity was expressed as mg CO_2_/kg·h.

#### 2.6.8 Vitamin C content

Vitamin C content according to a spectrophotometric method with minor modifications (29). Two gram sample was homogenized with 2 mL 1% pre-cooled HCl in an ice bath and the residue was washed three times with 1 mL 1% pre-cooled HCl. The above extracts were combined and transferred to a 10 mL brown centrifuge tube and centrifuged to obtain the supernatant (10,000 × g, 4°C, 20 min). 1 mL supernatant was added into a 10 mL brown volumetric flask containing 0.2 mL 10% HCl, and the volume was fixed with distilled water, the absorbance value at 243 nm was measured, and the Vitamin C content was calculated by ascorbic acid standard curve. The standard curve was shown in Supplementary Figure S2, and the equation of the standard curve was Y = 0.0457x + 0.0029.

#### 2.6.9 Total phenolic content

Total phenolic content was assayed in accordance with Folin–Ciocalteu method (29). 1 g sample was added with a little pre-cooled 1% HCL-methanol solution and ground into a homogenate under ice bath. The homogenate was filled with 1% HCL-methanol solution to 10 mL and extracted at 40°C in ultrasonic apparatus for 30 min, and then centrifuged (5,000 r/min, 30 min, 4°C). 0.5 mL the above supernatant and 1 mL 0.25 mmol /L Folin–Ciocalteu were reacted in the dark for 5 min, then 3 mL 7% Na_2_CO_3_ solution was added, and the volume was fixed to 10 mL with distilled water. After fully mixed, the absorbance was measured at 765 nm, and total phenolic content was calculated using the standard curve of gallic acid. The standard curve was shown in Supplementary Figure S3, and the equation of the standard curve was y = 0.0908x + 0.0369.

#### 2.6.10 MDA content

MDA content was detected with the thiobarbituric acid method (30). In brief, 1 g sample was added to pre-chilled trichloroacetic acid (1:5, w/v; 100 g/L) and homogenized in an ice bath. After centrifugation for 20 min (4°C 10,000 r/min), 2 mL crude extract was thoroughly mixed with a solution of thiobarbituric acid (1:1, v/v; 6.7 g/L). It was then boiled in a water bath for 20 min and centrifuged again. A solution of trichloroacetic acid (100 g/L) was used as a blank instead of the crude extract. Analyzing the supernatant absorbance at 450, 532, and 600 nm after the reaction resulted in nmol/g.

#### 2.6.11 H_2_O_2_ content

H_2_O_2_ content was determined according to the instructions of the commercial detection Kit (Solarbio Science & Technology Co., Ltd., Beijing, China). Each sample was weighed 0.1 g and performed three times in parallel.

#### 2.6.12 DPPH scavenging capacity

1 g sample was homogenized with 5 mL methanol and centrifuged (10,000 r/min, 15 min, 4°C) to obtain the supernatant. 0.1 mL the supernatant was mixed with 2.9 mL 0.1 mmol/L DPPH methanol and reacted for 30 min in the dark at 25°C. The methanol solution was used as a blank, and its absorbance was measured at 517 nm and recorded as A_1_, methanol instead of the supernatant was recorded as A_0_, and methanol instead of DPPH methanol was recorded as A_2_. The formula was calculated as follows:

$\mathrm{DPPH}\;\mathrm{scavenging}\;\mathrm{capacity}\left(\%\right)=\left(1-\frac{A_{1}-A_{2}}{A_{0}}\right)\times 100$

#### 2.6.13 Reducing power

For reducing power, the mixture contained 0.5 mL of the above methanol extract (1:5, w/v), 2.5 mL 0.2 mol/L sodium phosphate buffer (pH 6.6), and 2.5 mL 1% potassium ferricyanide solution. Place the mixture in a 50°C water bath for 20 min, add 2.5 mL 10% trichloroacetic acid (w/v) to terminate the reaction, and centrifuge (4,000 r/min, 15 min) to obtain the supernatant. Mix 2.5 mL supernatant and 2.5 mL distilled water with 0.5 mL 0.1% ferric chloride solution, and react at room temperature for 10 min. Measure its absorbance at 700 nm. The higher the absorbance, the stronger the reducing property.

#### 2.6.14 PPO activity

The sample was homogenized and extracted with 0.1 mol/L cold sodium acetate buffer (1:1, w/v, pH 5.5) containing 4% PVPP for 1 h at 4°C. The supernatant was centrifuged (10,000 r/min, 30 min, 4°C) and collected as enzyme extract for subsequent determination of PPO and POD activities. For the determination of PPO activity, 0.1 mL enzyme extract, 2.0 mL 0.1 mol/L pH 5.5 acetic acid–sodium acetate buffer, and 0.5 mL of 0.05 mol/L catechol solution were aspirated. The change in absorbance was recorded at 420 nm for 3 min, and a change of 0.01 in absorbance per min was defined as one PPO activity unit (U). The results were recorded as U/g.

#### 2.6.15 POD activity

In the case of POD activity, the reaction mixture consisted of 0.5 mL enzymatic extract, 3 mL 2% guaiacol, and 0.5 mL 0.5 mol/L H_2_O_2_ solution. The calculation of POD activity was based on the change in absorbance at 470 nm over 3 min, and a change of 0.01 in absorbance per min was defined as one POD activity unit (U). The results were recorded as U/g.

### 2.7 Statistical analysis

Three parallel experiments were conducted for each treatment, and data were presented as means ± standard. One-way analysis of variance (ANOVA) and Duncan’s multiple range test (*p* < 0.05) were used to compare between groups using SPSS version 22.0, and images were plotted using Origin 2021.

## 3 Results and discussion

### 3.1 Screening for optimal the ratio of nisin and ε-PL combined treatment

The effect of the ratio of nisin and ε-PL combined treatment on the total bacterial count of fresh-cut jackfruit is illustrated in Table 1. There was a gradual increase in total bacterial count for all treatment groups with prolonged storage, and combined treatment was significantly more effective than single nisin or ε-PL. When the combination ratio (nisin: ε-PL) is from 1:1 to 1:4, the antibacterial effect is continuously improved, which may be because ε-PL could penetrate microbial cells and cause interaction between ε-PL and nucleic acid to prevent nucleic acid transcription (31), resulting in microbial death. However, when the combination ratio was 1:5, the inhibitory effect was weakened instead. It is worth noting that the total bacterial count in the treatment group with a combination ratio of nisin to ε-PL of 1:4 was lower than all other treatment groups at 4 days and 8 days (*p* < 0.05), suggesting that this combination ratio had the best inhibitory effect and was used for the subsequent preservation of fresh-cut jackfruit.

**Table 1:** Effects of different ratios of nisin and ε-PL composite biological preservative on total bacterial count in fresh-cut jackfruit.

Storage time (d)	Treatment	Total bacterial count(logCFU/g)
0	–	2.30 ± 0.01
	Nisin	4.89 ± 0.06^a^
	ε-PL	4.64 ± 0.08^b^
	1:1	4.48 ± 0.05^c^
4	1:2	4.27 ± 0.06^d^
	1:3	4.22 ± 0.12^d^
	1:4	3.53 ± 0.02^e^
	1:5	4.20 ± 0.08^d^
	Nisin	5.27 ± 0.03^a^
	ε-PL	5.07 ± 0.05^b^
	1:1	4.90 ± 0.08^c^
8	1:2	4.66 ± 0.02^d^
	1:3	4.65 ± 0.05^d^
	1:4	4.36 ± 0.02^e^
	1:5	4.87 ± 0.05^c^

Different lowercase letters indicate differences between treatments at the same time point (*p* < 0.05).

### 3.2 Effects of nisin and ε-PL combined treatment on microorganisms

**Figure 1:**
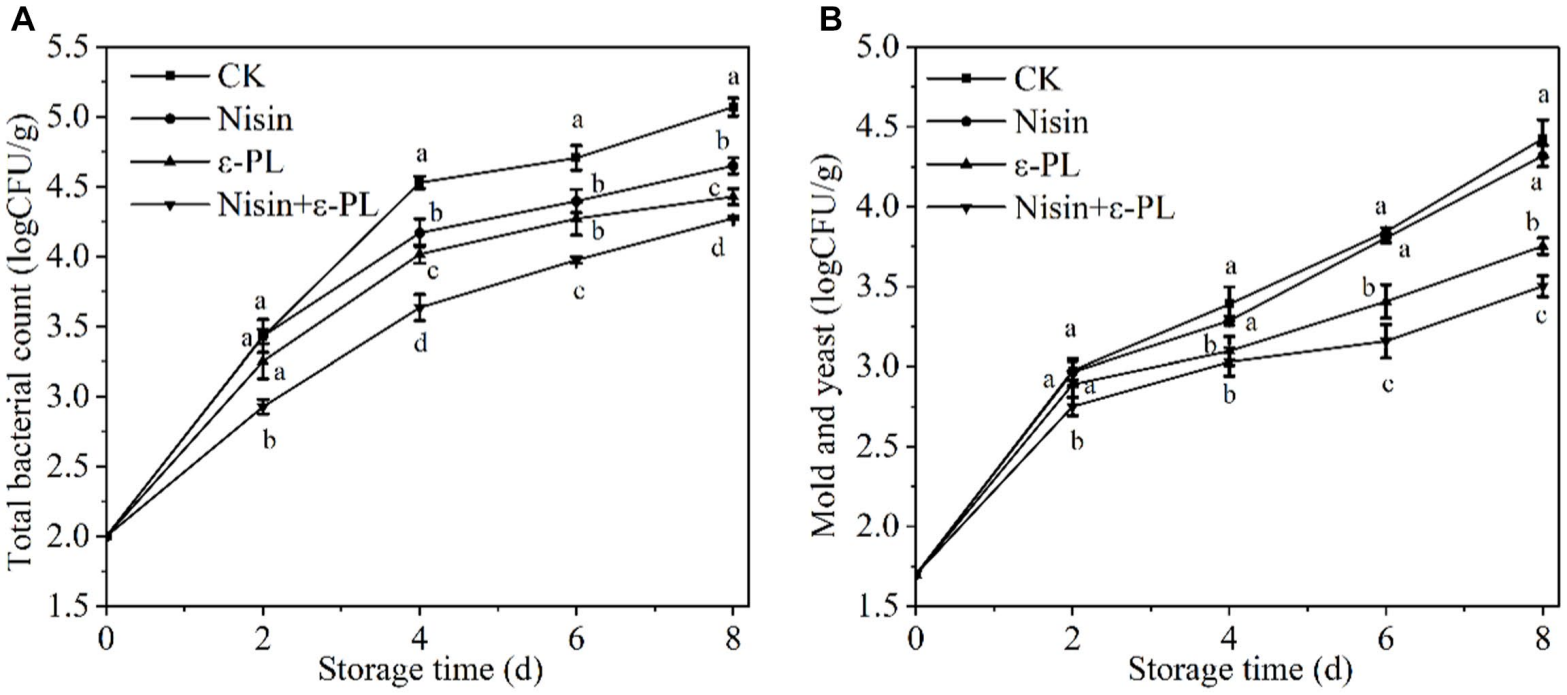
Effects of different treatments on total bacterial count **(A)**, mold and yeast **(B)** of fresh-cut jackfruit. All data are the mean values of three parallel experiments, and the vertical lines are the standard errors of mean values. Different lowercase letters (a–d) indicate significant differences between different treatment groups at the same time point (*p* < 0.05).

Fresh-cut fruit is more susceptible to microbial infestation, affecting its safety than intact fruit. Therefore, microbiological control in fresh-cut fruit is one of the most essential means to ensure quality. As shown in Figure 1A, the total bacterial count has steadily increased throughout storage. Each treatment group had a lower total bacterial count than CK group (*p* < 0.05), indicating that treatment with nisin or ε-PL contributed to delaying the increase of total bacterial count. Surprisingly, the nisin+ε-PL treatment was the lowest during the whole storage period (*p* < 0.05). This was mainly due to ε-PL altering cell membrane permeability, destroying cell structure, and inhibiting respiratory metabolism and enzyme activity, resulting in the interruption of cell matter, energy, and information transmission (32). Nisin rapidly destroyed cell structure and helped ε-PL enter the cell to inhibit microorganisms (33). Related literature has confirmed that nisin and ε-PL synergistically inhibit carrot microorganism growth (31).

According to Figure 1B, mold and yeast increased with time, following the same trend as the total bacterial count. A significant difference between nisin and CK was not observed during storage (*p* > 0.05), further confirming that nisin has a narrow antibacterial spectrum and has almost no inhibitory effect on Gram-negative bacteria, mold and yeast when used alone. The significant difference between ε-PL and CK during storage(*p* < 0.05), The mechanism of ε-PL inhibition of fungi can be attributed to the synergistic effects of antimicrobial actions such as electrostatic adsorption, plasma membrane hyperpolarization, and Accumulation of reactive oxygen species (ROS) (34). Mold and yeast in nisin+ε-PL treatment was lower than in a single treatment. Nisin plays a significant auxiliary antibacterial role, enhancing nisin+ε-PL treatment’s antifungal properties against mold and yeast. In conclusion, nisin+ε-PL treatment could effectively inhibit the increase in microorganisms and improve the quality.

### 3.3 Effects of nisin and ε-PL combined treatment on color

**Figure 2:**
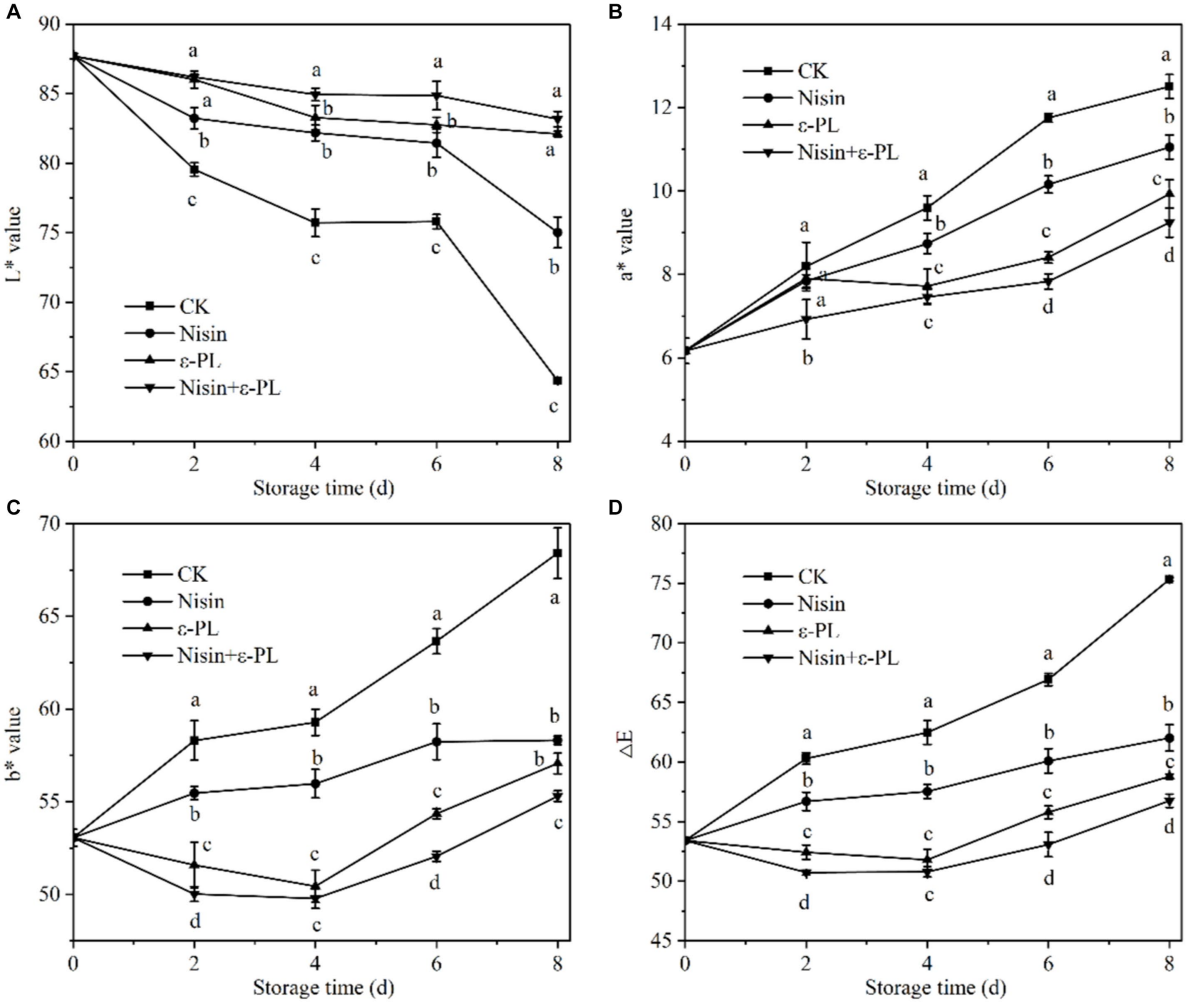
Effect of different treatments on L* value **(A)**, a* value **(B)**, b* value **(C)**, and △E **(D)** of fresh-cut jackfruit. All data are the mean values of three parallel experiments, and the vertical lines are the standard errors of mean values. Different lowercase letters (a–d) indicate significant differences between different treatment groups at the same time point (*p* < 0.05).

**Figure 3:**
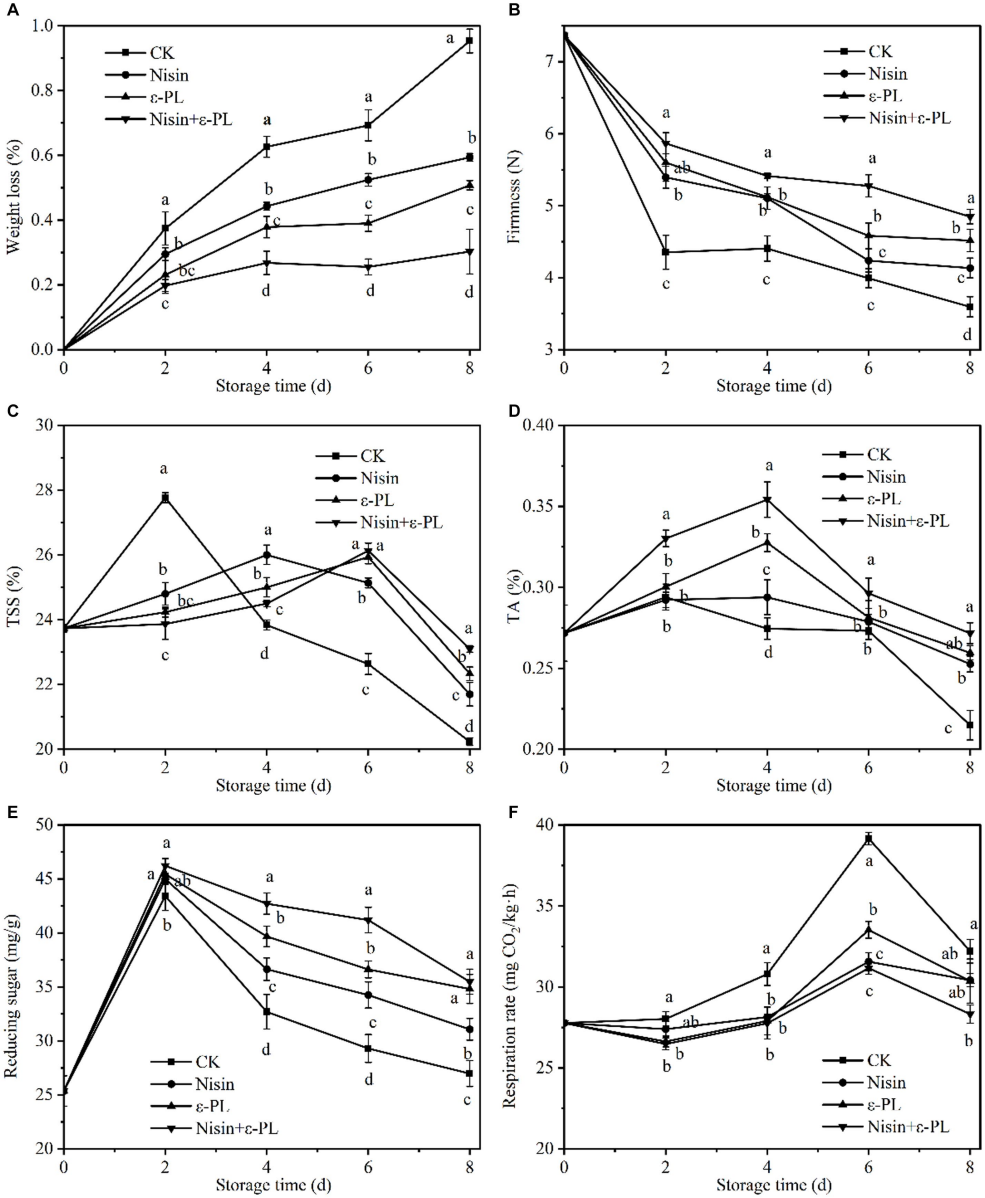
Effects of different treatments on weight loss **(A)**, firmness **(B)**, TSS **(C)**, TA **(D)**, reducing sugar **(E)**, and respiration rate **(F)** of fresh-cut jackfruit. All data are the mean values of three parallel experiments, and the vertical lines are the standard errors of mean values. Different lowercase letters (a–d) indicate significant differences between different treatment groups at the same time point (*p* < 0.05).

The color change is the most intuitive characteristic of the maturity and freshness of fresh-cut jackfruit, and it serves as an important parameter in consumer selection (35, 36). During storage, the L* value of all samples consistently decreases while a* value, b* value, and ∆E steadily increase. It can be seen from Figure 2A that during the entire storage period, the L* value of CK group was the lowest, followed by nisin and ε-PL, while the L* value of the nisin+ε-PL treatment was the highest, indicating that the nisin+ε-PL treatment could maintain brightness and delay browning of jackfruit during storage. As shown in Figure 3B, the a* value increases linearly, and the nisin+ε-PL treatment can maintain a low a* value. According to Figure 2C, both ε-PL and nisin+ε-PL treatments can maintain a low level of b* value, and b* value in group CK is as high as 68.41 at 8 days, while ε-PL and nisin+ε-PL treatments are only 56.31 and 55.30, respectively. As it may be gathered from Figure 2D, the ∆E change of nisin+PL treatment is the smallest, while the change of CK treatment is the largest, indicating that nisin+PL treatment had the best effect in delaying color changes. Studies have shown that fruit color change correlates with cell membrane semi-permeability. When the cell membrane is damaged, pigment material flows into the intercellular space along with intracellular fluids, altering the refractive index between the cell and its surrounding environment (37). In addition, enzymatic browning is the main factor causing browning in fresh-cut fruits and vegetables, and PPO and POD are key enzymes in enzymatic browning (38). Inhibiting enzyme activity can reduce enzymatic reactions and color changes. Nisin+ε-PL treatment can effectively inhibit microbial activities, maintain the integrity of the cell membrane, and reduce the flow of intracellular fluid and pigment material, and significantly reduce the activities of PPO and POD enzymes, thus maintaining the visual quality of fresh-cut jackfruit.

### 3.4 Effects of nisin and ε-PL combined treatment on weight loss, firmness, TSS, TA, reducing sugar, and respiration rate

As depicted in Figure 3A, all treatment groups showed a linear upward trend in weight loss. Compared with CK group, other treatment groups reduced weight loss to varying degrees, and nisin+ε-PL had the best effect. Nisin, ε-PL, and nisin+ε-PL treatment groups showed 0.34, 0.44, and 0.65% less weight loss than CK group at 8 days, respectively. The significant reduction in weight loss by nisin+ε-PL treatment may be due to the inhibition of microbial activity and maintenance of cell membrane integrity, thereby delaying water loss (39, 40), consistent with the above microbial results.

Due to physiological metabolism and water loss, fruit firmness will be visible and irreversible changes during fruit ripening, storage, and transportation (41). As can be seen from Figure 3B, in general, firmness of all samples decreased during storage, mainly related to cell wall composition, carbohydrate structure, and cell wall metabolic corresponding enzyme activity (42, 43). The initial firmness of fresh-cut jackfruit was 7.36 N, and the fruit firmness of CK group decreased the fastest during the storage period. The firmness retention effect was nisin+ε-PL>ε-PL>nisin>CK for the same storage period. The firmness of nisin+ε-PL treatment was 4.85 N at 8 days of storage, which was 34.72% greater than CK. The above findings implied that nisin+ε-PL is more suitable for maintaining fresh-cut jackfruit firmness.

TSS content is an essential indicator for measuring fruit maturity and intrinsic quality (44). As shown in Figure 3C, the TSS content in fresh-cut jackfruit was raised initially and declined subsequently during storage. The increase in TSS content is related to converting starches and other macromolecular carbohydrates into sugars by metabolic processes, transforming organic acids into sugars by gluconeogenesis, and increasing dry matter by water loss (45). The decrease in TSS content is due to the continual respiration of the fruit, which consumes more sugar than it produces. The peak value of CK group was 27.77% at 2 days of storage, then decreased sharply. The three treatment groups, nisin, ε-PL, and nisin+ε-PL, effectively delayed the arrival of the peak value.

As shown in Figure 3D, TA content increased first and then decreased with the ripening of fresh-cut jackfruit. At 4 days of storage, the ε-PL and nisin+ε-PL treatments peaked at 0.30 and 0.33%, respectively, significantly higher than CK and nisin treatments (*p* < 0.05). Some researchers have found that nisin is only effective against Gram-positive bacteria, and ε-PL has broad-spectrum inhibitory properties when nisin and ε-PL synergistically improve antibacterial activity and expand the inhibitory spectrum (46). Throughout the storage period, it was observed that the combination of nisin and ε-PL effectively preserved the titratable acid (TA) content. Previous research conducted by research (47) has demonstrated that the inhibition of microbial activity can effectively delay the degradation of titratable acids induced by microorganisms. The TA content is a good indicator of fruit maturation and senescence, and nisin+ε-PL treatment maintains a high titratable acidity content. This may be related to the fact that nisin+ε-PL inhibits respiration and maintains the integrity of cells.

Reducing sugar content was closely related to fruit maturity and storability. As shown in Figure 3E, the reducing sugar of all treatments exhibited an increasing trend at the beginning of storage, which may be caused by the hydrolysis of starch and other high-molecular carbohydrates. Subsequently, reducing sugar decreased, probably due to the vigorous metabolism of fresh-cut jackfruit consumed as a respiratory substrate (48). At 4–6 days of storage, all treatments were significantly higher than CK (*p* < 0.05). At the end of storage, the reducing sugar contents of CK, nisin, ε-PL, and nisin+ε-PL were 26.98 mg/g, 31.07 mg/g, 34.80 mg/g, and 35.48 mg/g, respectively, with the highest reducing sugar content in the nisin+ε-PL treatment. In addition, The research found that the decrease in reducing sugar was related to fungal spoilage (49), and the changing pattern of reducing sugar was consistent with the above microbial conclusion.

Respiratory metabolism is a crucial physiological activity associated with fruit ripening and senescence (50). From Figure 3F, fresh-cut jackfruit respiration rate increased and then decreased, and these changes were consistent with the patterns of TSS, TA, and reducing sugar quality indicators. As a climacteric fruit, fresh-cut jackfruit had a noticeable respiration peak when senescent. The treatment group did not delay the peak onset but significantly inhibited respiration rate (*p* < 0.05). In the early storage period, the treatment groups had no significant difference in respiration rate. Still, the nisin+ε-PL treatment’s respiration rate was the lowest at 8 days of storage.

### 3.5 Effects of nisin and ε-PL combined treatment on Vitamin C content, total phenolic content, MDA content, H_2_O_2_ content, the DPPH scavenging capacity, and reducing power

**Figure 4:**
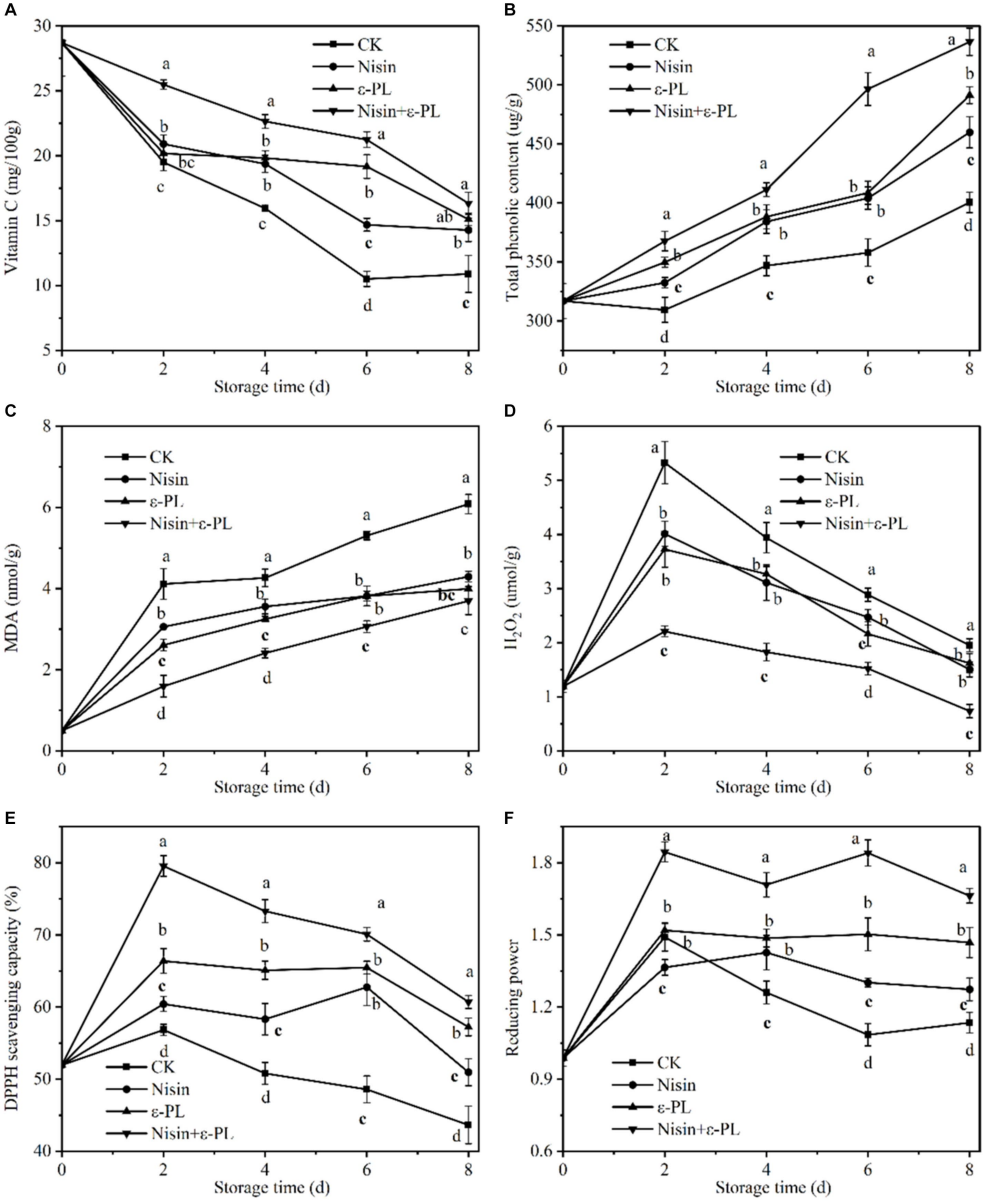
Effects of different treatments on Vitamin C content **(A)**, total phenolic content **(B)**, MDA content **(C)**, H_2_O_2_ content **(D)**, DPPH scavenging capacity **(E)**, and reducing power **(F)** of fresh-cut jackfruit. All data are the mean values of three parallel experiments, and the vertical lines are the standard errors of mean values. Different lowercase letters (a–d) indicate significant differences between different treatment groups at the same time point (*p* < 0.05).

Vitamin C content and stability are essential for fresh-cut fruit to maintain freshness, quality, and nutritional value, and its antioxidant property relieves tissue injury inflicted by free radicals and restrains bacterial infection (51). From Figure 4A, the Vitamin C content continuously decreased throughout the storage period, and all treatment groups were significantly higher than CK group (*p* < 0.05). Fresh-cut jackfruit treated with nisin+ε-PL had the best effect in keeping Vitamin C content. After storage for 8 days, Vitamin C content treated with nisin+ε-PL was 16.33 mg/100 g, which was 49.95% higher than CK group. This indicates that nisin+ε-PL treatment could effectively delay the decrease of Vitamin C content. In addition, research has found that nisin benefits antioxidant accumulation and helps pumpkins retain their quality (27), and ε-PL treatment maintains the Vitamin C content of fresh-cut lettuce (26).

Fruit’s nutritional value and storage stability are maintained by phenolics, which scavenge free radicals and retard lipid peroxidation. As illustrated in Figure 4B, the total phenolic content in all samples gradually increased due to the synthesis and accumulation of phenolic compounds induced by fresh-cut jackfruit wounds (52). Similar studies confirmed that fresh-cut strawberries (53) and fresh-cut pineapples (37) also showed an increasing trend during storage. During the whole storage period, the nisin+ε-PL treatment was always significantly higher than that of the single treatment (nisin or ε-PL) and CK group (*p* < 0.05). At the end of storage, the total phenolic content of nisin+ε-PL treatment was 536.54 μg/g, about 34% higher than that of CK group. Therefore, it can be speculated that nisin+ε-PL treatment can maintain a higher total phenolic content of fresh-cut jackfruit.

MDA is the end-product of lipid peroxidation, H_2_O_2_ induces oxidative damage in cells, and the level of both is an essential parameter for assessing membrane lipid peroxidation and cellular oxidative damage (54, 55). Figure 4C illustrates the change in fresh-cut jackfruit with time in MDA, which slowly increases over time. The MDA content of nisin, ε-PL, and nisin+ε-PL treatments were 4.29, 4.00, and 3.70 nmol/g at 8 days of storage, respectively, which were 30, 34, and 40% lower than that of CK group. This indicates that nisin+ε-PL treatment can reduce oxidative damage, maintain cell membrane integrity, and effectively inhibit the accumulation of MDA content.

As depicted in Figure 4D, the H_2_O_2_ content in all treatment groups exhibited a trend of initial increase followed by a subsequent decrease. The initial rise was attributed to the accumulation of ROS induced by mechanical cutting (56), which was subsequently reduced as the ripening and senescence of the fruit. Throughout the storage period, the H_2_O_2_ content of all treatment groups was significantly lower than that of CK group (*p* < 0.05), and there was no statistically significant difference between the nisin treatment and the ε-PL treatment, whereas the nisin+ε-PL could maintain a lower H_2_O_2_ content. The reason may be because nisin+ε-PL treatment held the higher antioxidant content of fresh-cut jackfruit. At the same time, it is accompanied by lower MDA content, which maintains cell membrane integrity, thus delaying senescence, improving oxidative stress capacity, and attenuating H_2_O_2_ accumulation.

DPPH scavenging capacity and reducing power are crucial indicators for assessing antioxidant capacity. Generally, reducing power is positively correlated with DPPH scavenging capacity (57). Therefore, when the DPPH scavenging capacity is higher, the reducing power tends to be higher as well. According to Figures 4E,F, the trends in DPPH scavenging capacity and reducing power were quite similar, showing an initial sharp increase followed by a gradual decline. From the beginning of storage to the end, it was observed that the DPPH scavenging capacity and reducing power of the nisin+ε-PL treatment significantly differed from those of CK group (*p* < 0.05). At 8 days of storage, the DPPH scavenging capacity and reducing power of the nisin+ε-PL treatment reached 60.70% and 1.66, respectively. In contrast, the corresponding values for CK treatment were 43.65% and 1.27, indicating that the nisin+ε-PL treatment could maintain a higher level of antioxidants. This effect may be attributed to the ability of nisin+ε-PL treatment to retard the loss of Vitamin C and maintain a higher total phenolic content, thereby enhancing the antioxidant capacity of fresh-cut jackfruit. Similar to our findings, cinnamon extract combined with ε-polylysine infusion increased the content of antioxidants such as ascorbic acid and total phenol (58).

### 3.6 Effects of nisin and ε-PL combined treatment on PPO and POD activities

**Figure 5:**
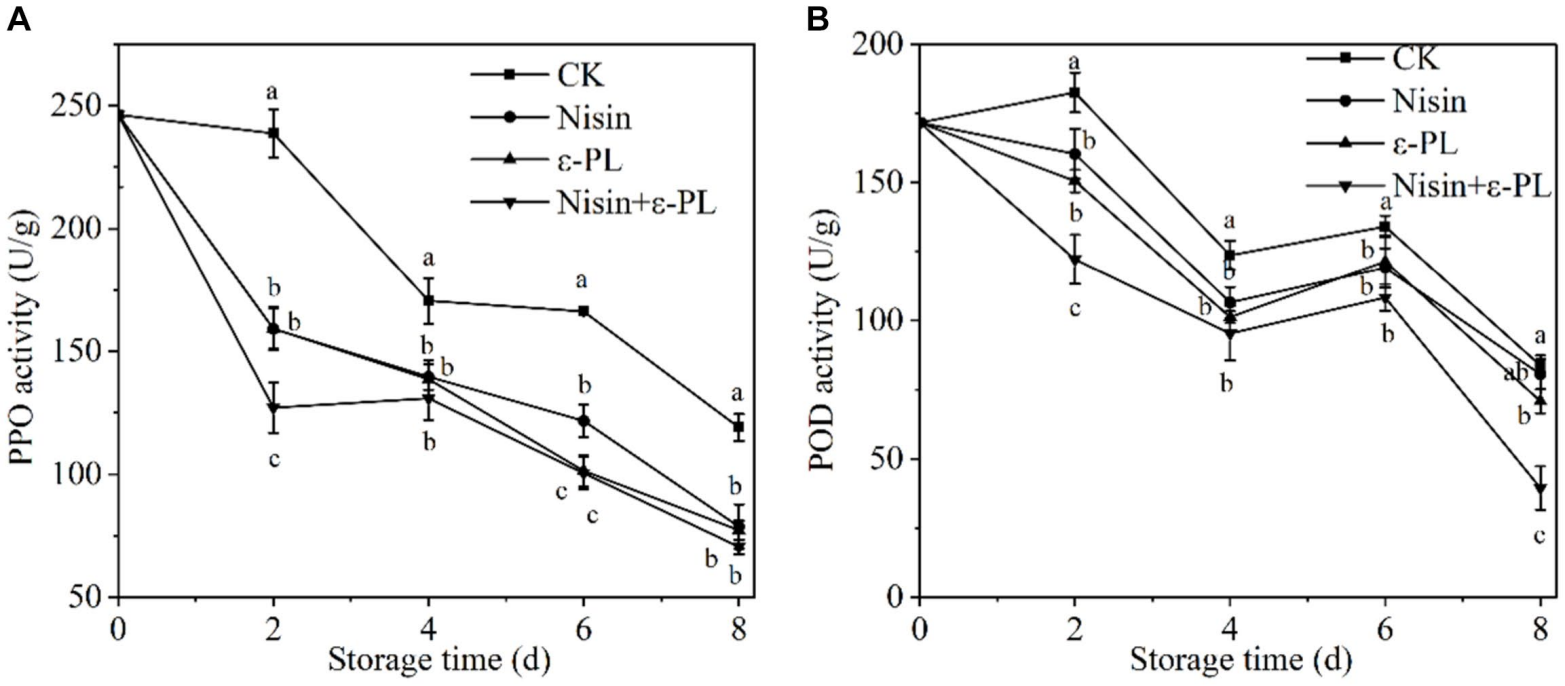
Effects of different treatments on PPO **(A)** and POD **(B)** activities of fresh-cut jackfruit. All data are the mean values of three parallel experiments, and the vertical lines are the standard errors of mean values. Different lowercase letters (a–d) indicate significant differences between different treatment groups at the same time point (*p* < 0.05).

PPO and POD are key enzymes responsible for fruit browning. Mechanical cutting disrupts the fruit tissue structure and cell spatial compartmentalization, and increases the possibility of contact between substrates and enzymes, thereby causing browning (59, 60). As can be seen from Figures 5A,B, the trends of PPO and POD activities were consistent with an overall decreasing trend. The enzymatic activity of jackfruit in CK group increased at 2 days, possibly due to the accumulation of H_2_O_2_ and oxidation of cell membranes caused by mechanical cutting, which stimulated the enhancement of PPO and POD activities. Throughout the storage period, PPO and POD activities in all treatment groups were significantly lower than those in CK group (*p* < 0.05), and the nisin+ε-PL treatment showed the lowest PPO and POD activities. The results indicated that nisin+ε-PL treatment was more beneficial in inhibiting the PPO and POD activities, significantly improving fresh-cut jackfruit’s storage quality and delaying its senescence and browning process.

## 4 Conclusion

As a typical climacteric fruit, jackfruit has vigorous postharvest physiological metabolism. Microorganisms easily infect it and accelerate physiological and biochemical changes after fresh cutting, which leads to quality deterioration and shortened shelf life of fresh-cut jackfruit. This article studied the effect of nisin combined with ε-PL on the microorganisms and refrigeration quality of fresh-cut jackfruit. The nisin and ε-PL combined treatment ratio was pre-screened through total bacterial count. When the ratio of nisin to ε-PL was 1:4, the best effect was found in inhibiting total bacterial count of fresh-cut jackfruit. The study found that compared with CK group, both nisin and ε-PL alone could inhibit the growth of total bacterial count, mold and yeast in fresh-cut jackfruit. However, nisin+ε-PL had the most significant inhibitory effect, which indicated that the two had a synergistic effect. In addition, nisin+ε-PL could better maintain the quality attributes of fresh-cut jackfruit, effectively delay the changes in the nutritional components of fresh-cut jackfruit during storage, reduce the weight loss rate, maintain a high firmness level and antioxidant capacity, and inhibit respiration rate, enzyme activities (PPO, POD) and MDA content increase, thereby significantly improving the storage quality of fresh-cut jackfruit.

## Data availability statement

The raw data supporting the conclusions of this article will be made available by the authors, without undue reservation.

## Author contributions

LZ: Formal analysis, Writing – original draft. AF: Formal analysis, Writing – original draft. GY: Supervision, Writing – review & editing, Funding acquisition. YN: Writing – review & editing, Data curation, Investigation, Methodology. YL: Data curation, Investigation, Methodology, Writing – review & editing. RY: Writing – review & editing, Supervision.
