# Supplementary material for: Nisin and ε-polylysine combined treatment enhances quality of fresh-cut jackfruit at refrigerated storage
Source: Front Nutr. 2024 Feb 14;11:1299810. doi: 10.3389/fnut.2024.1299810 (PMC10899680; doi:10.3389/fnut.2024.1299810)
Supplement: Supplementary file 1 [file Data_Sheet_1.docx]

**Nisin and ε-polylysine combined treatment enhances quality of fresh-cut jackfruit at refrigerated storage**

Liping Zeng ^1, 2^, Aiping Fan^1, 2^, Guangming Yang ^1, 2^, Yuping Nong^1^, Yifan Lu ^1^, Ruopeng Yang ^1, 2 *^

^1^ College of Chemistry and Resources Engineering, Honghe University, Mengzi, 661100, People's Republic of China

^2^ Yunnan Province International Joint Laboratory of Green Food, College of Chemistry and Resources Engineering, Honghe University, Mengzi, Yunnan, 661100, China


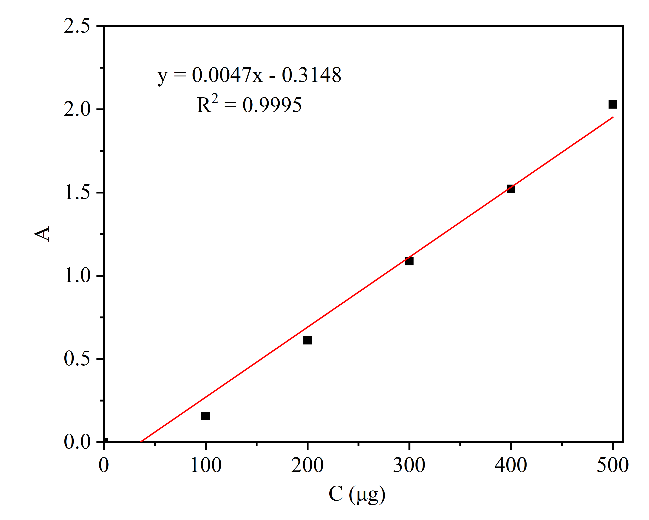


**Figure S1.** Calibration curve of absorbance versus glucose concentration


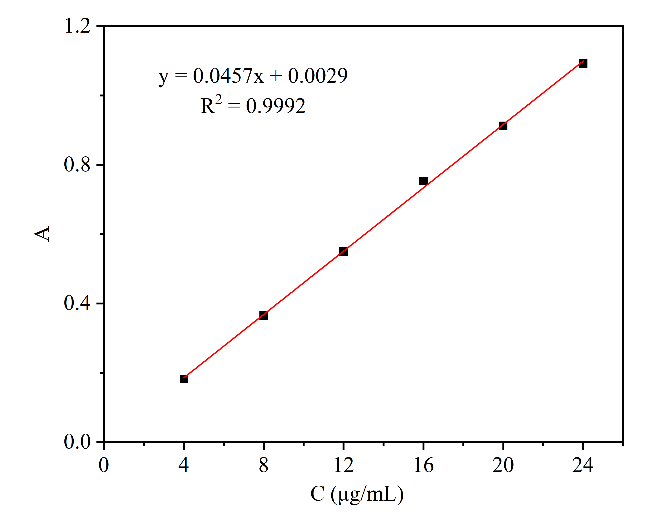


**Figure S2.** Calibration curve of absorbance versus ascorbic acid concentration


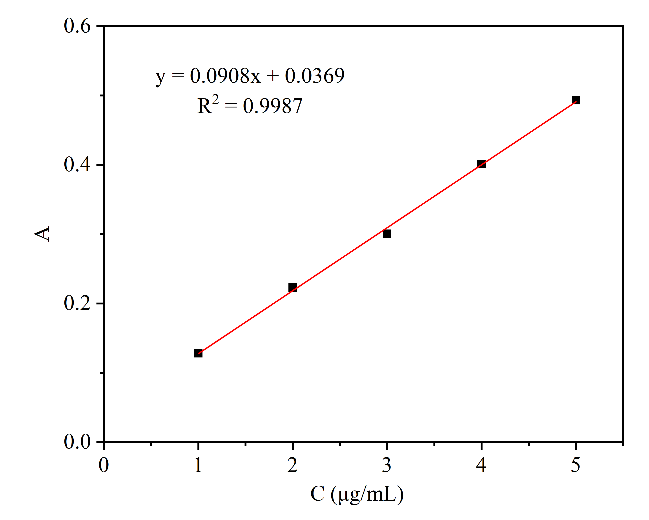


**Figure S3.** Calibration curve of absorbance versus gallic acid concentration
